# Comparative analysis of clinical characteristics of COVID-19 among vaccinated and unvaccinated patients in a major treatment facility in Ghana

**DOI:** 10.4314/gmj.v57i4.6

**Published:** 2023-12

**Authors:** Benedict NL Calys-Tagoe, Joseph Oliver-Commey, Georgia NK Ghartey, Abdul G Mohammed, Delia Bandoh, Christian Owoo, Ernest Kenu

**Affiliations:** 1 Department of Community Health, University of Ghana Medical School, Korle Bu, Accra; 2 Ghana Infectious Disease Centre, Ga East, Accra; 3 Ghana Field Epidemiology and Laboratory Training Programme; 4 Department of Anaesthesia, University of Ghana Medical School, Korle Bu, Accra; 5 Department of Epidemiology and Disease Control, University of Ghana School of Public Health, Legon, Accra

**Keywords:** SARS-CoV-2, vaccination status, Ghana, COVID-19, COVAX, GIDC, Ga East

## Abstract

**Objectives:**

To compare clinical characteristics of COVID-19 among vaccinated and unvaccinated patients in a major treatment facility in Ghana.

**Design:**

A retrospective study drawing on data from COVID-19 patients' records visiting the facility from March 2021 to December 2021.

**Setting:**

Ghana Infectious Disease Centre, Ga East Municipality, Greater Accra Region, Ghana.

**Participants:**

In-patients and outpatients who reported to the facility from 1st March 2021 to December 2021 were included in the study, and patients with missing data on vaccination were excluded.

**Outcome measures:**

underlying conditions, symptoms, case management information, hospital service rendered (OPD, HDU or ICU), length of hospital stay, treatment outcome

**Results:**

The study included 775 patient records comprising 615 OPD and 160 hospitalised cases. Less than one-third (26.25%; 42) of the patients hospitalised were vaccinated compared to almost 40.0% (39.02%; 240) of the patients seen at the OPD. Vaccinated individuals were nearly three times (aOR = 2.72, 95%CI:1.74-4.25) more likely to be managed on an outpatient basis as compared to the unvaccinated. The death rate among the vaccinated group and the unvaccinated were (0.71%; 2) and (3.45%; 17), respectively, with a significant reduction in the risk of dying among the vaccinated compared to the unvaccinated (aOR = 0.13, 95%CI: 0.028 0.554).

**Conclusions:**

Less than half of the in-patient and OPD patients were vaccinated. Mild infections, fewer days of hospitalisation, outpatient treatment and higher chances of survival were associated with being vaccinated against SARS-CoV-2. Prudent measures should be implemented to encourage the general public to take up SARS-CoV-2 vaccines.

**Funding:**

None declared

## Introduction

As of May 20^th^, 2022, the Corona Virus Disease 2019 (COVID-19) caused by the Severe Acute Respiratory Syndrome Coronavirus 2 (SARS-COV 2) had led to about 522 million infections and 6 million deaths world-wide.^1^ Since the index case was reported in Ghana in March 2020, the country has recorded about 161,000 cases, resulting in about 1,500 deaths.^2^

About 4 billion people in the world have been fully vaccinated with the various types of COVID-19 vaccine since the vaccines were first rolled out in December 2020.^3^ As of May 18^th^, 2022, 6 million persons in Ghana had been fully vaccinated via the COVID-19 Vaccines Global Access (COVAX) initiative.^2^,^4^,^5^

This notwithstanding, the COVID-19 vaccination coverage in the country is relatively lower than the WHO immunisation target rate of 70% of the country's total population.^2^,^6^ Also, a study on vaccine hesitancy reported that approximately 40.7% of Ghanaians were reluctant to receive the vaccine, with concerns about its efficacy, side effects and association with infertility.^7^ Studies have shown the association of vaccination with reducing morbidity and mortality rates and disease severity in other parts of the world.^8^-^10^

However, there is still a dearth of information and documentation of the disease's clinical characteristics among vaccinated and non-vaccinated, specific to the Ghanaian population. This study aimed to compare the clinical characteristics of COVID-19 (including the resulting severity of disease) among vaccinated and unvaccinated patients in a major treatment facility in Ghana.

## Methods

**Study Design**: We conducted a retrospective study among COVID-19 cases reporting to the Ghana Infectious Disease Center, Ga East. Data on both COVID-19 in-patients and outpatients from March 2021 to December 2021 were obtained from the health facility.

**Study Setting**: The Ghana Infectious Disease Center (GIDC) is a 100-bed capacity facility located in the Ga East Municipality of the Greater Accra Region. The GIDC was commissioned on July 24^th^, 2020, to serve as a National Case Management Coordinating Treatment Centre for the clinical management of COVID-19 and, eventually, other infectious diseases. The facility runs an Outpatient Department (OPD) and a Post-COVID-19 Clinic, with the capacity to admit patients to its High Dependency (HDU) and Intensive Care Units (ICU). The clinical staff of the centre includes an infectious disease specialist, an anaesthesiologist/intensivist, 15 medical officers, 40 nurses, and 13 laboratory personnel. As of May 2022, 1004 walk-in (OPD) cases and 317 admissions have been attended at the treatment centre. However, only patients who reported to the facility after vaccination against COVID-19 had begun in the country -after 1^st^ March 2021, were included in this study.

**Data collection:** Data on COVID-19 patients reporting to the GIDC (Outpatient clinic and Admission wards) from March 2021 to December 2021 was extracted from patient folders using a digital abstraction form on the KoboCollect version 2021.2.4 platform. All patients with missing data on vaccination were excluded from the study.

**Data Variables**: Data extracted for analysis comprised demographic information (age, sex, occupation, marital status and residence), epidemiologic information (travel history, epidemiological links), clinical information (vital signs, underlying conditions, symptoms, case management information, hospital service rendered (OPD, HDU or ICU), length of hospital stay if admitted and treatment outcome). Also, data were extracted on patients' COVID-19 vaccination (status, vaccine type, and regimen completion). Fully vaccinated patients were defined as those with a full series of any COVID-19 vaccine at least 7 days before hospitalisation. Partially vaccinated were those with an incomplete series of any COVID-19 vaccine, and unvaccinated were those with no COVID-19 vaccine.^11^ Disease severity information was also abstracted from patient folders. Cases were classified as mild, moderate, severe or critical based on the WHO criteria for classifying COVID-19 cases.^12^

**Data analysis**: The data extracted were cleaned and imported into StataIC 15 (StataCorp, College Station, TX, USA) for analysis. Categorical variables were expressed as frequencies and percentages with their corresponding 95% CI. Parametric continuous variables were expressed as means and standard deviation. The chi-square test of association and ANOVA were used to compare the clinical characteristics of the vaccinated and unvaccinated patients. Binary logistic regression analysis was performed to test the association between vaccination status and the various clinical characteristics. Variables significant at a P-value < 0.25 at the unadjusted level were selected and fitted into an adjusted logistic model. The P-value < 0.25 used in the adjusted model allowed for the inclusion of more explanatory variables in the adjusted model. The level of significance for the final model was set at 5%.

### Ethical considerations

Ethical clearance for this study was sought from the Ghana Health Service Ethics Review Committee (GHS-ERC 006/05/20). The administration of the GIDC formally granted permissions. Throughout the data abstraction and analysis, generated codes were used in place of personal identifiers of patients to ensure patient anonymity and preserve confidentiality. Individual patient consent was not obtained for the study as the data was accessed retrospectively and strictly anonymised.

## Results

### Characteristics of the Patients Studied

A total of 856 patient records were available, of which 81 had missing information on vaccination status and were thus excluded from the analysis. Hence, analysis was done on 775 patient records comprising 615 OPD and 160 hospitalised cases. The median age of in-patients was 55.0 (IQR 36.0 – 67.0) years. Most hospitalised patients were males (51.88%; 83), whilst most patients attended to at the OPD were females (55.61%; 324). More than half (60.63%; 97) of the patients admitted had co-morbidities, whilst less than one-third(25.05%; 154) of those attended to at the OPD were with co-morbidities. On the severity and outcome of the infection, almost two-thirds (62.50%; 100) of the patients admitted were severely or critically ill, while none of the patients seen at OPD had a severe or critical illness. Almost 12% (11.88%; 19) of the patients admitted and managed died compared to zero mortality recorded among the patients seen at OPD. Regarding the vaccination status among the patients, less than one-third (26.25%; 42) of the patients hospitalised were vaccinated against COVID-19, while almost 40.0% (39.02%; 240) of the patients seen at the OPD were vaccinated (Table 1).

**Table 1:** Characteristics of study participants

Characteristics	In-Patient	Outpatient
		
	**n (%)**	**n (%)**
**Sex**		
**Female**	77 (48.12)	342 (55.61)
**Male**	83 (51.88)	273 (44.39)
**Age (median; IQR) years**	55.0 (IQR 36.0 – 67.0)	33.0 (IQR 26.0 – 44.0)
**<25 years**	14 (8.75)	121 (19.67)
**25 – 34 years**	19 (11.88)	208 (33.82)
**35 – 44 years**	24 (15.00)	133 (21.63)
**45 – 54 years**	21 (13.12)	76 (12.36)
**55+ years**	82 (51.25)	77 (12.52)
**Marital status**		
**Single**	29 (18.13)	134 (21.79)
**Married**	54 (33.75)	168 (27.32)
Not stated**	77 (48.12)	348 (56.59)
**Co-morbidity**		
**No**	63 (39.37)	496 (80.65)
**Yes**	97 (60.63)	154 (25.05)
**Hospitalisation care**		
**High Dependency Unit**	147 (91.88)	-
**Intensive Care Unit**	13 (8.12)	-
**Initial severity of disease**		
**Mild**	26 (16.25)	582 (94.63)
**Moderate**	34 (21.25)	33 (5.37)
**Severe/Critical**	100 (62.50)	0 (0.00)
**Median Length of Hospitalization**	16.00 days (IQR 10.00 - 24.00)	-
**Treatment outcome**		
**Alive**	141 (88.12)	615 (100.00)
**Dead**	19 (11.88)	0 (0.00)
**Vaccination status**		
**Unvaccinated**	118 (73.75)	375 (60.98)
**Vaccinated**	42 (26.25)	240 (39.02)
**Vaccination completion**		
**Partial**	28 (66.67)	174 (72.50)
**Complete**	14 (33.33)	66 (27.50)
**Reported Type of vaccine**		
**AstraZeneca**	17 (40.48)	150 (62.50)
**J&J**	2 (4.76)	21 (8.75)
**Pfizer**	3 (7.14)	14 (5.83)
Not stated**	20 (47.62)	55 (22.92)

IQR: Interquartile Range; n: absolute numbers.

**Distribution of COVID-19 infection and Vaccination status among patients** More than half of all those infected with COVID-19 (54%) and those vaccinated (57%) were females. The 25 – 34 age group was the most affected (29.29%; 227) and the most vaccinated (31.56%; 89). (Table 2).

**Table 2:** Distribution of COVID-19 infection and Vaccination among patients

Variables	COVID 19 infection	Vaccinated
	**n (%)**	**n (%)**
**Sex**		
**Female**	419 (54.06)	161 (57.09)
**Male**	356 (45.94)	121 (42.91)
**Age**		
**<25 years**	135 (17.42)	29 (10.28)
**25 – 34 years**	227 (29.29)	89 (31.56)
**35 – 44 years**	157 (20.26)	56 (19.86)
**45 – 54 years**	97 (12.52)	34 (12.06)
**55+ years**	159 (20.52)	74 (26.24)
**Marital status**		
**Single**	163 (42.34)	41 (31.30)
**Married**	222 (57.66)	90 (68.70)
**Co-morbidity**		
**No**	468 (65.09)	158 (60.77)
**Yes**	251 (34.91)	102 (39.23)

n: absolute numbers

### Association between vaccination status and clinical presentation among COVID-19 patients

A multivariate logistic regression analysis revealed a statistically significant association between case category, length of hospitalisation, care setting, treatment outcome and vaccination status. Vaccinated individuals were nearly three times (aOR = 2.72, 95%CI:1.74-4.25) more likely to be managed on an outpatient basis as compared to the unvaccinated.

The death rate among the vaccinated group and the unvaccinated were (0.71%; 2) and (3.45%; 17), respectively, with a significant reduction in the risk of dying among the vaccinated compared to the unvaccinated (aOR = 0.13, 95%CI: 0.028-0.554). Compared to unvaccinated patients, those vaccinated had reduced severe disease states (6.38% vs 16.63%). However, the severity of the disease was not significantly associated with vaccination status (Table 3).

**Table 3:** Association between vaccination status and clinical presentation among COVID-19 patients

Variables	Unvaccinated n(%)	Vaccinated n(%)	cOR (95%CI)	aOR (95%CI)
**Case category**				
In-patient	118 (23.94)	42 (14.89)	Ref	Ref
OPD	375 (76.06)	240 (85.11)	1.80 (1.220 - 2.650)	2.72 (1.740 - 4.250)
**Disease severity**				
Mild	372 (75.46)	236 (83.69)	Ref	Ref
Moderate	39 (7.91)	28 (9.93)	1.19 (0.701 - 2.005)	0.79 (0.453 - 1.390)
Severe/Critical	82 (16.63)	18 (6.38)	0.35 (0.203 - 0.593)	0.18 (0.098 - 0.322)
**Care Setting**				
High Dependency Unit/ Intensive Care Units	118 (23.94)	42 (14.89)	Ref	Ref
Home treatment	375 (76.06)	240 (85.11)	1.90 (1.2736 - 2.844)	2.80 (1.802-4.354)
**Median Length of Stay in the Hospital**	22.52 (±12.705)	8.07 (±5.048)	0.68 (0.598 -0.783)	0.68 (0.590-0.781)
**Treatment Outcome**				
Alive	476 (96.55)	280 (99.29)	Ref	Ref
Death	17 (3.45)	2 (0.71)	0.21 (0.048 - 0.915)	0.13 (0.028-0.554)

cOR: Crude Odds Ratio; aOR: Adjusted Odds Ratio; CI: Confidence Interval; Ref: Reference; n: absolute numbers

### Association between vaccination status and patient-reported symptoms among COVID-19 patients

Patient data with missing vaccination status were excluded from the analysis of the reported symptoms. Regarding patient-reported symptoms, the loss of taste (14.15% vs 76.08%), dyspnoea (13.16% vs 90.91%), loss of smell (17.17% vs 27.00%) and chest pains (29.03% vs 88.10%) were all reduced in the vaccinated group compared to the unvaccinated. A multivariable logistic regression analysis controlling for age, sex, marital status, occupation, and co-morbidities revealed a statistically significant association between patients' vaccination status and various symptoms such as loss of taste, dyspnoea, palpitations, insomnia, chest pains and loss of smell. The odds of palpitation report among those vaccinated was reduced by 98% compared to the unvaccinated (aOR = 0.02, 95%CI: 0.006 0.038). The study also revealed a 94% reduction in the odds of loss of taste reported among those vaccinated compared to their counterparts (aOR = 0.06, 95%CI: 0.027 0.154). There was also a 91% reduction in the odds of dyspnoea among the vaccinated group compared to the unvaccinated (aOR = 0.09, 95%CI: 0.028 0.271) (Table 4).

**Table 4:** Association between vaccination status and patient-reported clinical symptoms among COVID-19 patients

Variables	Unvaccinated	Vaccinated	cOR (95%CI)	aOR (95%CI)
	n (%)	n (%)		
**Fever**				
**No**	233 (66.76%)	156 (74.64%)	Ref	Ref
**Yes**	116 (33.24%)	53 (25.36%)	0.68(0.465-1.001)	0.41 (0.106-1.599)
**Loss of Taste**				
**No**	100 (23.92%)	176 (85.85%)	Ref	Ref
**Yes**	318 (76.08%)	29 (14.15%)	0.05(0.033- 0.081)	0.06 (0.027-0.154)
**Cough**				
**No**	152 (40.97%)	83 (37.56%)	Ref	Ref
**Yes**	219 (59.03%)	138 (62.44%)	1.15 (0.820-1.624)	1.55 (0.831-2.888)
**Dizziness**				
**No**	176 (76.86%)	98 (80.33%)	Ref	Ref
**Yes**	53 (23.14%)	24 (19.67%)	0.81 (0.473-1.398)	1.37 (0.627-2.970)
**Dyspnoea**				
**No**	35 (9.09%)	99 (86.84%)	Ref	Ref
**Yes**	350 (90.91%)	15 (13.16%)	0.02 (0.008-0.029)	0.09 (0.028-0.271)
**Palpitations**				
**No**	18 (5.54%)	38 (76.00%)	Ref	Ref
**Yes**	307 (94.46%)	12 (24.00%)	0.02(0.008-0.041)	0.02 (0.006-0.038)
**Fatigue**				
**No**	152 (59.14%)	102 (65.38%)	Ref	Ref
**Yes**	105 (40.86%)	54 (34.62%)	0.77(0.507-1.158)	0.83 (0.361-1.915)
**Headache**				
**No**	144 (49.15%)	77 (46.39%)	Ref	Ref
**Yes**	149 (50.85%)	89 (53.61%)	1.12 (0.763-1.635)	1.37 (0.652-2.879)
**Myalgia**				
**No**	93 (62.84%)	56 (56.57%)	Ref	Ref
**Yes**	55 (37.16%)	43 (43.43%)	1.30 (0.773-2.181)	1.32 (0.622-2.803)
**Insomnia**				
**No**	58 (16.76%)	65 (87.84%)	Ref	Ref
**Yes**	288 (83.24%)	9 (12.16%)	0.03(0.013-0.059)	0.20 (0.004-0.093)
**Loss of Smell**				
**No**	219 (73.00%)	164 (82.83%)	Ref	Ref
**Yes**	81 (27.00%)	34 (17.17%)	0.56 (0.358-0.878)	0.35 (0.136-0.912)
**Chest pain**				
**No**	55 (11.90%)	154 (70.97%)	Ref	Ref
**Yes**	407 (88.10%)	63 (29.03%)	0.06 (0.037-0.083)	0.05 (0.027-0.094)

cOR: Crude Odds Ratio; aOR: Adjusted Odds Ratio; CI: Confidence Interval; Ref: Reference; n: absolute numbers

## Discussion

In the global fight against the COVID-19 pandemic, vaccination has been reported to impact infection severity and disease outcomes in countries where vaccines have been introduced. This study compared the clinical characteristics of COVID-19 among vaccinated and unvaccinated patients at the GIDC in a bid to contribute to studying the significance of vaccination against COVID-19 in the Ghanaian setting,

For hospitalised and OPD cases reviewed in this study, more than 60% of the patients who presented with the infection had not been vaccinated as of December 2021. This is similar to the findings of other studies that indicate that the fewer vaccinated cases are due to heightened immunity and reduced susceptibility to the infection in individuals, which is offered by vaccination.^13^–^16^ Our study differs from other studies where cases were higher among vaccinated people because of breakthrough infections after vaccination and underlying conditions.^17^,^18^

It is worth noting that many of the vaccinated patients had been vaccinated with AstraZeneca, a double-dose course vaccine. This vaccine was the first to be received in Ghana and has been the most used for vaccination against COVID-19 in the country so far.^19^ In this study, more than half of patients who had been vaccinated had not undertaken the full vaccination regimen, as was seen in other studies where it was found that low vaccine supplies, hesitancy and lack of adequate information on vaccination schedule influenced incompleteness.^20^,^21^ Studies have shown that some form of protection was offered to individuals, whether partially or fully vaccinated.^22^

From this study, being vaccinated decreased the odds of requiring admission to high-dependency or intensive care units for severe disease. The Appropriateness Evaluation Protocol (AEP) confirms that the hospital service received by a patient indicates disease severity, with OPD cases having a less severe form of the disease.^23^ Hence, this study's finding is similar to others where vaccination was seen to reduce hospitalisation rates, explained based on the elicited immune response after vaccination.^22^,^24^,^25^ In this same vein, being vaccinated decreased the odds of dependency on hospital care (HDU/ICU) when an individual was down with the disease because the severity in such patients was lower compared to patients who had not been vaccinated. However, in countries with much higher coverage, there were larger numbers of vaccinated patients with higher disease severity because the base population at risk of being infected largely included already vaccinated people.^26^ This study manifests that vaccination decreased the odds of lengthy admission days among patients who had been admitted.

This could be due to the relatively faster resolution of patients' symptoms and reduced need for specialised care and organ support, as a similar Norway study showed.^27^ However, in another study, there was no significant difference between the length of hospital stay among vaccinated and unvaccinated patients, which was said to have been influenced by the fact that the patients being studied were geriatric and had common underlying conditions.^28^ In addition, this study further demonstrates that being vaccinated decreased the odds of death among patients on admission, suggesting reduced disease severity and its associated indicators among vaccinated patients.

Our study reports reduced odds of reporting ageusia, dyspnoea, palpitations, insomnia, anosmia and chest pain among vaccinated patients compared to those who had not been vaccinated. Among these symptoms are palpitations, insomnia and dyspnoea, which are indicators of higher severity of the disease.^29^ However, there was no significant difference in the reporting of fever, cough, dizziness, fatigue, headache symptoms, and myalgia, which are much milder symptoms present in both vaccinated and unvaccinated patients.^29^

Though using data from a single facility in this study could be a limitation for generalising our findings, this centre is currently the only infectious disease centre in the country and receives referrals from other treatment centres. Therefore, the findings in this study are seemingly essential in telling the Ghanaian story.

## Conclusion

Less than half of both the in-patient and OPD patients were vaccinated. Mild infections, fewer days of hospitalisation, outpatient treatment and higher chances of survival were associated with being vaccinated against SARS-CoV-2. Also, there were reduced odds of reporting ageusia, dyspnoea, palpitations, insomnia, anosmia and chest pain among vaccinated patients compared to those who had not been vaccinated. These findings support the claim that vaccination against COVID-19 reduces disease severity and the risk of dying. Therefore, prudent measures should be implemented to encourage the general public to take up SARS-CoV-2 vaccines.
